# Cardiovascular genetics: technological advancements and applicability for dilated cardiomyopathy

**DOI:** 10.1007/s12471-015-0700-y

**Published:** 2015-06-02

**Authors:** G.J.M. Kummeling, A.F. Baas, M. Harakalova, J.J. van der Smagt, F.W. Asselbergs

**Affiliations:** 1 Department of Cardiology, Division Heart and Lungs, University Medical Center Utrecht, Room E03.511, PO Box 85500, 3508 GA Utrecht, The Netherlands; 2 Department of Medical Genetics, University Medical Center Utrecht, Utrecht, The Netherlands; 3 Durrer Center for Cardiogenetic Research, ICIN-Netherlands Heart Institute, Utrecht, Utrecht, The Netherlands; 4 Faculty of Population Health Sciences, Institute of Cardiovascular Science, University College London, London, United Kingdom

**Keywords:** Genetics, Cardiology, Cardiomyopathy, Dilated, Review

## Abstract

Genetics plays an important role in the pathophysiology of cardiovascular diseases, and is increasingly being integrated into clinical practice. Since 2008, both capacity and cost-efficiency of mutation screening of DNA have been increased magnificently due to the technological advancement obtained by next-generation sequencing. Hence, the discovery rate of genetic defects in cardiovascular genetics has grown rapidly and the financial threshold for gene diagnostics has been lowered, making large-scale DNA sequencing broadly accessible. In this review, the genetic variants, mutations and inheritance models are briefly introduced, after which an overview is provided of current clinical and technological applications in gene diagnostics and research for cardiovascular disease and in particular, dilated cardiomyopathy. Finally, a reflection on the future perspectives in cardiogenetics is given.

## Introduction

### Genetic variants, mutations and inheritance models

The inheritance of various traits or diseases from parent to offspring occurs primarily through DNA. When comparing two random human individuals, their DNA is approximately 99.9 % identical. The remaining 0.1 % of DNA is responsible for their differences (variations)[1]. The DNA of an individual can change or become mutated in several ways, ranging from small-scale variants that affect just one or a few nucleotides (small substitutions, insertions and deletions) [2] to large-scale alterations affecting the chromosome structure, for example with copy number variations or translocations[3].

In the case of a nucleotide change, the term ‘variant’ often refers to a mere difference compared with a reference genome (which is publically accessible online). The term ‘mutation’ is more used for changes which cause impairment of protein function and lead to disease[4]. Mutations exist in various types, each with differing likelihood to be damaging. Types that generally produce large effects are for example stop-gain (also called nonsense), essential splice site and frameshift mutations, which usually cause early stops, thereby impairing protein formation, leading to an incomplete or excessively large protein. Though not necessarily damaging, these types of mutations understandably have the potential to severely impair biological function. Other so-called missense mutations more subtly change one amino acid (which are the building blocks of proteins), while leaving the rest of the protein intact. Hence, these mutations are less likely to be severely damaging. Constitutions of nucleotides at a certain place in a gene are called alleles: for example at a certain place in a chromosome, one individual has allele A and the other individual has allele T. Since, in humans, two copies exist of every chromosome, individuals can be homozygous for an allele (existing on both chromosomes, e.g. AA, CC, GG, TT) or heterozygous (existing on one chromosome, e.g. AC, AG, AT, CG, CT, GT). Individuals carrying only one copy of an allele or chromosome are hemizygous (for example the X-chromosome in males).

The first basic genetic inheritance models date back from the nineteenth century, when Gregor Mendel, an Augustinian monk, posed rules for dominant and recessive inheritance. These rules still constitute the basis of current views on certain inheritance models of human diseases, [5] which are nowadays referred to as ‘Mendelian’ models. Another present-day example reflecting Mendel’s merits is the Online Mendelian Inheritance in Man website (OMIM, via http://www.omim.org), which is a widely used online catalogue of human genes and genetic disorders [6]. Clinical examples in this article will be provided with a so-called MIM number, which can be used to access more related information on the OMIM website.

Mendel’s theory on inheritance mainly comprised two models: dominant and recessive. In an autosomal dominant inheritance pattern, a certain phenotype is caused in a heterozygous individual by a single, dominantly acting allele. This allele can be transferred to the offspring with a 50 % chance for both females and males (e.g. *MYH7* mutations in dilated cardiomyopathy, MIM#613426) [7]. In autosomal recessive diseases, two alleles have to be mutated for (full) manifestation of the phenotype. This can be either at the same position in a gene in homozygous individuals (often caused by related, consanguineous parents) [8] or at two different positions in a gene, in so-called compound heterozygous individuals (with one allele maternally, the other allele paternally inherited) [9]. In recessive disease, heterozygous carriers are usually healthy or only mildly affected. Sex-linked diseases follow different rules than autosomal diseases. Since males receive only one maternal X-chromosome (and therefore are hemizygous) they are more likely to be affected by a mutation on the X-chromosome. Females have two X-chromosome copies, so mutations in one chromosome can be (partially) compensated by the healthy allele on the other X-chromosome [10]. Y-linked diseases naturally appear only in males [11].

## Clinical genetics

### General introduction

Clinical genetics comprises the diagnostic process of, and adaptation to the medical, psychological and familial implications of genetic disease.[12] Various medical specialists refer a paediatric or adult patient (proband/index) with a suspected genetic disease to outpatient genetics clinics for evaluation. Medical history (including prenatal, neonatal period) of motor and intellectual development, physical examination for growth parameters, dysmorphic features (unusual morphology of the face and body) and pedigree construction (diagram representing how a trait or disease is segregating in a family) are important factors to assess the possibility for a genetic cause of the disease. Diagnostic differentials are established by appraising clinical information and consulting online databases. These contain medical photos and extensive phenotypic information to provide assistance in pinpointing the right diagnosis in the ‘jungle’ of rare genetic diseases (e.g. OMIM, London Medical Database—LMD).[6]

After examination, genetic diagnostic tests can be performed. Examples of genetic tests are DNA sequencing (reading parts of DNA) of candidate genes (e.g. *MYBPC3* in dilated cardiomyopathy, MIM#615396) [12], karyotyping and array analysis (e.g. for investigating chromosomal abnormalities, such as Down syndrome or 22q11 deletion syndrome) [13]. If family members of the index patient are concerned about the risk of developing the same disease themselves or in a second child, genetic counselling with risk assessment, presymptomatic testing, prenatal diagnostics or sometimes even preimplantation genetic screening (genetic profiling of embryos) can be offered [14]. Clinical geneticists often collaborate with research groups when they are unable to identify the underlying genetic defect of the patient’s disease. This mainly involves additional sequencing of unexplored regions of the patient’s DNA.[15]

### Clinical applicability in dilated cardiomyopathy

**Fig. 1:**
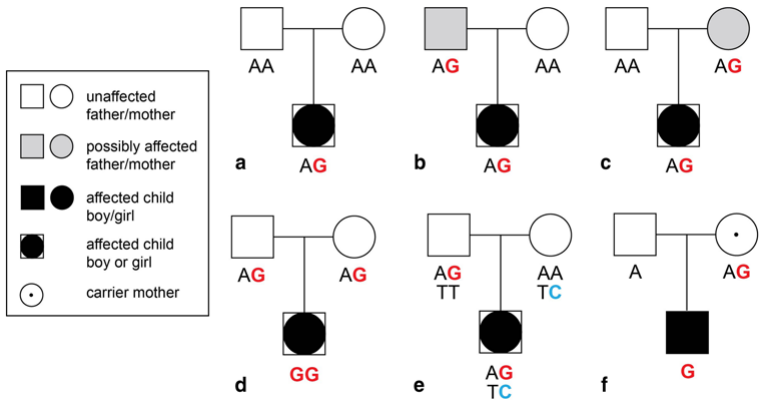
Examples of inheritance in pedigrees with seemingly sporadic cases: **a** de novo, **b**/**c** autosomal dominant with reduced penetrance (= a mutation does not consequently cause disease), **d**: autosomal recessive, **e**: compound heterozygous (two mutations in one gene, yet in two different alleles, collectively constituting an effect), **f** X-linked inheritance

Genetic counselling can be relevant for dilated cardiomyopathy (DCM) patients, since the disease has been demonstrated to frequently have a genetic origin, even in seemingly sporadic cases—that is, DCM patients with no self-reported family history of cardiomyopathy. In 1992, Michels et al. showed by screening family members of DCM patients with echocardiography that DCM is heritable in at least one in five patients. The majority of family members were asymptomatic with only a dilated left ventricle, and some developed symptoms in the following years. Since DCM can have such presymptomatic stages whereby index patients appear to be sporadic, routine family screening with echocardiography of family members of DCM patients is recommended (Fig. 1 **)** [16]. In the case of familial DCM, genetic testing can subsequently be considered, particularly with accompanying conduction disorder(s) and/or arrhythmia [17]. The beneficial role of genetic diagnostics in truly sporadic idiopathic DCM has not been empirically substantiated yet. In general, cardiac genetic disorders are characterised by a heterogeneous background, with variable penetrance and expressivity. This means that clinical phenotypes can vary within families, even when pedigrees share the same mutation. Hence, one can find mutation carriers having no clinical manifestations of the disease to having severe disease with various manifestations [18].

The diagnosis of genetic cardiomyopathies can be beneficial at multiple clinical levels. In diagnostics and therapy, presymptomatic investigation (mainly for family members) could lead to possible health benefit by early treatment of disease. The potential of early treatment for mutation carriers is currently being assessed by the PRECARDIA trial. Here, an angiotensin-converting enzyme inhibitor (ACE inhibitor) is administered to family members of DCM patients that carry a (presumed) pathogenic mutation, to see if this can delay or prevent the occurrence of DCM [19]. In 2006, Meune et al. performed implantable cardiac defibrillator (ICD) implantation in patients with a lamin A/C (*LMNA*) mutation, who were only in need of a pacemaker. They showed effectiveness in treating possibly lethal tachyarrhythmias in 42 % of the patients, with an appropriate shock percentage of 89 %. Of note, improvement of survival could not be assessed, since the study did not contain a control group [20]. Another example of therapeutic consequences is lifestyle recommendations for patients with hypertrophic cardiomyopathy, such as abstinence from top sports [21]. Also patients with arrhythmogenic right ventricular dysplasia/cardiomyopathy are advised against practising competitive and endurance sports, for arrhythmia prevention [22].

In prognostics, Van Spaendonck-Zwarts et al. demonstrated that the prevalence of mortality, heart transplantation and malignant ventricular arrhythmias was higher among DCM patients with a mutation in the phospholamban (*PLN)* or *LMNA* gene, compared with DCM patients who did not carry a mutation in diagnostically screened genes [23]. Of note, mutations in these two genes can also manifest themselves clinically. A deletion of arginine 14 in the PLN gene can, for example, cause dilated cardiomyopathy with attenuated electrocardiographic (ECG) R amplitudes or low voltages in multiple leads on the ECG [24, 25]. *LMNA* mutations in turn, typically show a low amplitude P wave and prolongation of the PR interval with a narrow QRS complex on the ECG. Furthermore, patients with these gene mutations are at higher risk to develop conduction disorders and/or arrhythmias.[26]

### Technological advancements

In the last decade, DNA sequencing in genetic diagnostics has undergone some tremendous changes. Due to technological advancements with next-generation sequencing (NGS), the capacity and cost efficiency of DNA sequencing has grown enormously by creating the ability to screen multiple genes simultaneously [27]. Before NGS, Sanger sequencing was the standard method in clinical laboratories to screen for mutations. Though very reliable, this method has smaller throughput, meaning that usually one gene was screened at a time, and that the maximum number of genes to be screened was also practically limited. Since many genes are involved in the pathogenesis of cardiomyopathies (DCM for example has more than 45 implicated genes), NGS subsequently provides an enormous potential for improvement in terms of efficiency in cardiovascular genetic testing, when compared with Sanger sequencing.[28]

The discovery rate in cardiovascular genetics has correspondingly grown rapidly. In 2012, for example, using NGS, Herman et al. discovered the important role of the titin (*TTN*) gene in the disease aetiology of dilated cardiomyopathy [29]. After its successful debut in research, NGS has been implemented over the last years in multiple diagnostic centres. Here, multiple genes can be screened simultaneously in multiple patients on a single chip (so-called gene panel). Currently, 45 genes are being sequenced in multiple centres across the Netherlands. These genes are implicated in highly diverse biological structures and pathways, among which sarcomere integrity, ion channels, metabolism, calcium regulation and transcriptional control [30–32]. Alongside its application in diagnostics, NGS is sometimes deployed in research settings to screen for mutations in any desired combination of genes, or even all genes simultaneously. The latter is called whole exome sequencing and is mainly suitable for cases with large families available for genetic screening, or for patients in which *de novo* (arose in the genome after fertilisation) mutations are expected based on the pedigree. In that case, trio analysis is performed, in which the whole exome sequence of a patient is compared with that of the (healthy) parents.

With NGS, earlier shortcomings of Sanger sequencing in terms of throughput and cost efficiency have certainly been overcome. Now the genetic community is facing a whole new challenge though, which is adequately interpreting the significance of identified variants. Genetic variants are frequent, and most are known to be harmless. The more genes are investigated, the more variants one will find. With the capacity of NGS to harvest so many variants, effective means to differentiate between innocent and pathogenic variants are essential [33].

**Fig. 2:**
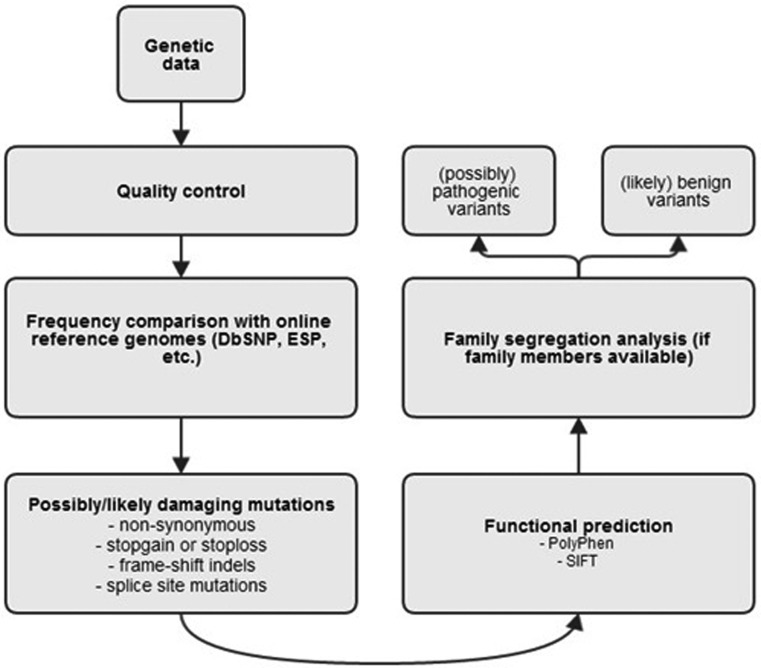
Global overview of genetic data analysis, by filtering for multiple criteria: (i) rare variants (by comparison with online reference genomes such as the Single Nucleotide Polymorphism DataBase (*dbSNP*), or Exome Sequencing Project (*ESP*)), (ii) mutation effect (previously explained) (iii) functional prediction (*SIFT* Sorting Intolerant From Tolerant), the likelihood of variants to be damaging is assessed

Besides the obvious optimisation of data quality to ensure actual presence of a variant, the likelihood of variants to be pathogenic is assessed in various ways (Fig. 2). First, one can assess the frequency of a certain variant in the general population in order to distinguish rare from common variants, by comparison with online reference genomes. The underlying idea here is that common variants cannot be causal for rare diseases [34].

Second is mutation effect assessment. Here only variants that potentially impair protein structure and/or function are selected (these mutation types are explained above, under ‘introduction’). Third, the likelihood of missense and splice-site variants to be damaging is assessed with software programmes (SIFT and PolyPhen) that predict possible impact on the structure and function of a human protein using straightforward physical and comparative considerations [35].

Fourth, after this initial workup of identified variants, an important analysis step is family segregation, which is considered the cornerstone of genetic counselling. It basically comprises genetic screening of affected family members, to see if a mutation segregates with disease. The stronger a mutation segregates with disease, the more likely it is to be causal. Fifth is functional follow-up, where mutations are investigated, in for example animal models, to assess their biological effect. Due to time and cost constraints, functional tests are unfortunately not available for diagnostics, and therefore are only occasionally deployed in genetic research.

The analysis methods described above can reduce the number of suspicious variants to a great extent. Nonetheless we are often left with a high number of so-called variants of uncertain significance (VUS), impeding effective diagnostics. Hence, for patients with idiopathic dilated cardiomyopathy, the advent of NGS has cleared up the genetic cause for some patients, while generating tremendous uncertainty for many patients with its high number of VUSses. This means that, in the anticipation of genetic screening, one must consider carefully how many genes are to be screened. The principle applies that when fewer genes are screened (with a so-called ‘targeted approach’), less variants will likely be detected, thus facilitating their interpretation—at the same time though, sensitivity diminishes, meaning that finding no variant in a subset of genes does not exclude a genetic cause. Inversely, simply choosing a broad approach with whole exome sequencing will often result in an unsolvable wealth of variants, with a low specificity. The uncertain role of variants and their pathogenicity is even shown to extend to variants previously deemed to be causal for DCM. In 2013, Andreasen et al. showed, by using online mutation databases and large publicly available reference genome datasets, that ‘known DCM-causing variants’ were in fact more prevalent than estimates of DCM prevalence. This justly questioned the pathogenicity of these variants and even raised the question whether the prevalence of DCM is higher than previously estimated [34]. This was later shown to be likely by Hershberger et al. [32] using various estimation methods.

## Future perspectives

Regarding current developments in the field of cardiovascular genetics, several issues will likely be addressed more adequately in the foreseeable future, of which a selection will be highlighted here.

In diagnostics, an obvious issue that needs to be addressed is the high number of VUSses. An important strategy to tackle this is data sharing. By sharing variants with their associated phenotypes on publicly accessible databases (e.g. GENCOR, via http://www.durrercenter.nl/catalogue), the mass of cardiogenetic evidence will be more accessible. This will allow an efficient gain of knowledge and better interpretation of variants in the future. Another important development in disputing the numerous VUSses is enhanced data interpretation with multi-variant analysis. Here, distinction is made between disease causing variants and so-called effect modifiers, which enables a more adequate, nuanced interpretation of the role of variants in pathogenesis of DCM. This was recently shown by, for example, Roncarati et al. [36], who discovered in an extended family with DCM the causal role of a *LMNA* mutation, with a disease-aggravating mutation in *TTN*. Another development in diagnostics is preimplantation genetic diagnostics, a technique which allows for genetic testing of an embryo, before its implant (e.g. in-vitro fertilisation or intracytoplasmatic sperm injection) [37].

For therapy, numerous efforts are being undertaken in the field of genetics to treat or prevent cardiac disease. In pharmacogenetics, genetic profiling is used as a determinant for genotype-guided personalised medicine.[38] In the RAPID GENE trial (ReAssessment of Anti-Platelet Therapy Using an InDividualized Strategy Based on GENetic Evaluation), point-of-care genetic testing was used to identify CYP2C19*2 carriers, which is an allele associated with increased rates of major adverse events. After randomisation, genetic testing proved to be an effective deciding factor to reduce high on-treatment platelet reactivity which, in turn, is associated with a lower complication rate. [39]

Another promising treatment option is gene therapy. Currently, SERCA2a gene therapy trials are ongoing to treat patients with heart failure. Several studies have demonstrated a decrease of sarco-endoplasmic reticulum calcium-ATPase 2a (SERCA2a) expression and function in heart failure patients. In phase II of the Calcium upregulation by percutaneous administration of gene therapy in cardiac disease (CUPID) trial, an adeno-associated virus is used to deliver SERCA2a to the cardiomyocytes. After 12 months, patients with high dose showed a decrease in heart failure symptoms, increased functional status and reversal of the negative left ventricle remodelling [40]. In another trial involving SERCA2a gene expression modification in a mouse model, Wahlquist et al. [41] described how they improved survival for heart failure in a mouse model by injecting an antagonist for micro-RNA 25. Potentially in 2015, the first gene therapy approved for clinical use will be rolled out (Glybera®, for treatment of lipoprotein lipase deficiency) [42]. Further, research will ultimately tell to what extent gene therapies are suitable as a treatment for heart failure or DCM, and if cardiogenetics will live up to its potential promise in the context of personalised medicine.

## Concluding remarks

In the last decade, cardiovascular genetics has undergone tremendous progression, mainly thanks to the implementation of NGS. Hence, the pathophysiological insight into, among other diseases, dilated cardiomyopathy is increasing and modernised genetic testing has quickly been integrated in clinical practice. The main challenges currently reside on the area of improving variant interpretation and possibly, the application of gene (regulation) therapy to treat and prevent dilated cardiomyopathy and other cardiovascular disorders. With its boosted discovery rate and increasingly prominent clinical role, this quickly emerging field of medicine is expected to continue to have great impact on cardiovascular healthcare.
